# Anti-Arrhythmic Effects of Sodium-Glucose Co-Transporter 2 Inhibitors

**DOI:** 10.3389/fphar.2022.898718

**Published:** 2022-06-24

**Authors:** Yuling Jing, Ruixue Yang, Wen Chen, Qiang Ye

**Affiliations:** Department of Cardiology, The Affiliated Hospital of Southwest Medical University, Luzhou, China

**Keywords:** arrhythmia, SGLT2I, cardiac electrical remodeling, mitochondrial dysfunction, oxidative stress, cardiac structural remodeling, inflammation

## Abstract

Arrhythmias are clinically prevalent with a high mortality rate. They impose a huge economic burden, thereby substantially affecting the quality of life. Sodium-glucose co-transporter 2 inhibitor (SGLT2i) is a new type of hypoglycemic drug, which can regulate blood glucose level safely and effectively. Additionally, it reduces the occurrence and progression of heart failure and cardiovascular events significantly. Recently, studies have found that SGLT2i can alleviate the occurrence and progression of cardiac arrhythmias; however, the exact mechanism remains unclear. In this review, we aimed to discuss and summarize new literature on different modes in which SGLT2i ameliorates the occurrence and development of cardiac arrhythmias.

## Introduction

Arrhythmia is a common cardiovascular disease (CVD) generally associated with organic heart diseases. Different types of arrhythmias are known where bradyarrhythmias and tachyarrhythmias are the most common. The development of pacemakers has proven to be a boon for patients with bradyarrhythmias. However, the onset of tachyarrhythmia is usually acute, and some tachyarrhythmias, such as atrial fibrillation (AF), ventricular tachycardia, and ventricular fibrillation (VF), increase the risk of disability and death (Gheini et al., 2020). Although radiofrequency ablation has been shown to improve the health of patients with tachyarrhythmias (Morillo et al., 2014; Guandalini et al., 2019), the overall therapeutic effect is still inadequate, especially the lack of drugs for tachyarrhythmias. This insufficiency is because the currently available ones pose a risk of developing arrhythmias (Tisdale et al., 2020).

Sodium-glucose cotransporter 2 inhibitors (SGLT2is) are a new class of hypoglycemic drug that includes empagliflozin (EMPA), dapagliflozin (DAPA), canagliflozin (CANA), sotagliflozin, and ertugliflozin. Unlike traditional hypoglycemic drugs, this class does not rely on improving insulin secretion or resistance. However, it reduces the renal threshold for glucose and increases its excretion in the urine by inhibiting sodium-glucose cotransporter 2 of the proximal renal tubules from active reabsorption of glucose, thus lowering blood glucose (Zelniker and Braunwald, 2020). This anti-diabetic mechanism is weakened at low blood glucose concentrations, thereby reducing the risk of hypoglycemia. The DECLARE-TIMI 58 trial showed that the incidence of hypoglycemic events was significantly lower in the dapagliflozin group than in the control group (Wiviott et al., 2019). Additionally, SGLT2i also plays a role in lowering blood pressure, lipid regulation, anti-inflammatory response, and lowering of uric acid levels (Chino et al., 2014; Hayashi et al., 2017; Tentolouris et al., 2019; Arow et al., 2020). A large number of studies have confirmed that SGLT2i also plays a significant role in cardioprotection (Zelniker et al., 2019a; Zelniker et al., 2019b; Wiviott et al., 2019). The DECLARE-TIMI 58 trial showed that the cardiorenal secondary composite outcome in patients with type 2 diabetes mellitus (T2DM) was significantly reduced with dapagliflozin compared to placebo (hazard ratio = 0.76, 95% CI = 0.67–0.87; *p* < 0.0001) (Mosenzon et al., 2019). The DAPA-HF, EMPEROR-Reduced, and EMPA-REG OUTCOME trials have confirmed that SGLT2i can significantly reduce the occurrence and progression of heart failure (HF) and cardiovascular events (Zelniker et al., 2019a; McMurray et al., 2019; Zannad et al., 2020). Recently, SGLT2i has been reported to improve arrhythmias. The DECLARE-TIMI 58 trial showed that DAPA reduced the risk of atrial fibrillation/atrial flutter (AF/AFL) by 19% in patients with type 2 diabetes (Zelniker et al., 2020). In mice with mitral regurgitation, DAPA reduced the induction rate and duration of AF (Lin et al., 2021). EMPA was reported to shorten the Q-T interval of diabetic rats (QTc: 190 ± 4 ms vs. 160 ± 3 ms, *p* < 0.005) and reduce the duration of action potential (Lee et al., 2019). Additionally, it did not change the heart rate or QTc interval in patients with T2DM, but QTc dispersion could be significantly reduced (Sato et al., 2017). In diabetic mice, EMPA affected the inter-atrial conduction time and a high dose (30 mg/kg) reduced the incidence of AF from 85% to 36.8% (Shao et al., 2019). It also reduced the induction rate of VF post myocardial ischemia (Azam et al., 2021). However, the specific mechanism by which SGLT2i ameliorates arrhythmias remains unclear. Arrhythmias are related to cardiac electrical remodeling, mitochondrial dysfunction, oxidative stress, cardiac structural remodeling, inflammation, and autophagy. Based on the above-mentioned factors, we aimed to review the possible mechanisms of SGLT2i in improving arrhythmia conditions.

### Cardiac Electrical Remodeling and Arrhythmias

Arrhythmia is closely related to cardiac electrical remodeling. In 1995, Wijffels et al. found that in the goat model of AF created by implanting a pacemaker, the atrial effective refractory period gradually shortened with the extension of atrial pacing time. This proved that cardiac electrical remodeling is involved in the process of AF and that cardiac electrical remodeling caused AF to persist (AF-induced AF) (Wijffels et al., 1995). Ion channel remodeling is the basis of cardiac electrical remodeling. Notably, intracellular Na^+^ and Ca^2+^ overload, caused by the Na^+^ and Ca^2+^ channel remodeling, and the disorder of K^+^ currents, play important roles in the occurrence and maintenance of arrhythmias.

The late sodium current (*I*_Na-Late_) is formed by a small amount of slow inactivation or reopening of sodium channels during myocardial repolarization, persisting in the repolarization phase of the action potential, especially the plateau phase, and is important for regulating the duration of action potentials (Zaza et al., 2008). Its duration was 10–100 ms, and its amplitude was approximately 1% of the peak sodium current (peak *I*_Na_). When *I*_Na-Late_ increases, the increased Na^+^ influx causes Na^+^ overload in cardiomyocytes (Wu et al., 2009; Sossalla et al., 2010; Poulet et al., 2015; Zhang et al., 2017), which increases the incidence of arrhythmias. Studies have shown that delayed afterdepolarizations (DADs), early afterdepolarizations, and continuous triggering activity occur with the persistence of Anemonia sulcata toxin II. Both DADs and continuous triggering activity decreased after the administration of ranolazine and tetrodotoxin, suggesting that inhibiting *I*_Na-Late_ could ameliorate arrhythmias (Belardinelli et al., 2006; Song et al., 2008). The increased activity of the Na^+^-H^+^ exchanger (NHE) also plays an important role in the occurrence and maintenance of arrhythmias (Ayoub et al., 2007; Hui et al., 2008; Altemose et al., 2001). NHE is essential for the regulation of Na^+^ concentration in cardiomyocytes (Madonna and De Caterina, 2013). In a canine AF model, the NHE-selective inhibitor HOE642 significantly improved the effective refractory period, thereby improving AF. This can be attributed to the reduction in the Na^+^ concentration in cardiomyocytes (Jayachandran et al., 2000).

Under physiological conditions, L-type Ca^2+^ channels (I_CaL_)of cardiomyocyte membrane are activated in the depolarization phase. Thus, a small amount of Ca^2+^ enters into the cytoplasm and binds to Ca^2+^-binding sites in the sarcoplasmic reticulum (SR) membrane. This is followed by opening of Ca^2+^-releasing channels ryanodine receptors 2 (RYR2) in SR leading to generation of the Ca^2+^ spark. This results in an increase of intracellular Ca^2+^ to over one hundred times instantly and is referred to as calcium-induced calcium release (CICR) (Fabiato, 1983; Cannell and Kong, 2017). Ca^2+^ sparks are the basis for excitation-contraction coupling in cardiomyocytes (Cheng et al., 1993). Na^+^-Ca^2+^ exchanger (NCX) is a type of membrane protein involved in bidirectional transport (3 Na^+^ for 1 Ca^2+^) and is regulated by a transmembrane Na^+^ gradient. In the case of Na^+^ overload, NCX reverse transport leads to cytoplasmic Ca^2+^ increase, eventually leading to Ca^2+^ overload. Increased Ca^2+^ levels can induce CICR, enhance the excitability of cardiomyocytes, and increase the possibility of arrhythmias (Greiser and Schotten, 2013; Chu et al., 2019). Ca^2+^ leakage from SR is also responsible for Ca^2+^ overload in cardiomyocytes. Hyperphosphorylation of RYR2 induced by the increased activity of Ca^2+^/calmodulin-dependent protein kinase type-II (CaMKII) is the basis of Ca^2+^ leakage from the SR in cardiomyocytes of patients with arrhythmias (Sag et al., 2009; Neef et al., 2010; Voigt et al., 2012). Studies have reported that *I*_Na-Late_ influences Ca^2+^ homeostasis in cardiomyocytes by activating CaMKII (Fischer et al., 2015). In addition, sarcoplasmic/endoplasmic reticulum Ca^2+^ ATPase 2a (SERCA2a), a calcium pump on SR, plays an important role in maintaining intracellular Ca^2+^ homeostasis in cardiomyocytes (Zhang et al., 2018). During myocardial diastole, most of the Ca^2+^ in the cytoplasm of cardiomyocytes is recycled to the SR via SERCA2a. When the quantity and activity of SERCA2a decreases, the amount and rate of Ca^2+^ clearance in the cytoplasm is affected, leading to Ca^2+^ overload and therefore arrhythmias (Lyon et al., 2011; Zaman et al., 2016; Samuel et al., 2018).

The K^+^ currents are the main current in the cardiomyocytes’ repolarization phase, they have been confirmed to be related to arrhythmias, including the transient outward K^+^ current (I_to_), the delayed-rectifier K^+^ current (I_K_), the inward rectifier K^+^ current (I_K1_), and the ATPsensitive K^+^ current (K_ATP_) (Tamargo et al., 2004). The I_to_ plays a major role in the reduced gradient in action potential duration (APD) (Volk et al., 2001). Cardiomyocytes’ APD will be prolonged with the density of Ito decreasing. Yue et al. found that in the AF dog model, the decreasing of the I_to_ was related to altering cardiac electrophysiology and promoting arrhythmia maintenance (Yue et al., 1999). And the I_K_ is the main current in the repolarization phase 3 of cardiomyocytes in many animals. It is closely related to the duration of cardiomyocytes’ APD and effective refractory period (ERP). The risk of sudden cardiac death may be enhanced by the decreasing of I_K_ (Lengyel et al., 2008)_._ I_K1_ is the most important current to maintain and stabilize the cardiomyocytes’ resting potential, and modulate the final repolarization phase of the action potential (AP), thus exerting profound effects on cardiac excitability and arrhythmogenesis. Studies reported I_K1_ is closely with ventricular arrhythmias after myocardial infarction (Zhai et al., 2017; Zhai et al., 2019).

### SGLT2i Ameliorates Electrical Remolding

SGLT2i have been shown to improve intracytoplasmic Na^+^ overload in cardiomyocytes. Philippaert found that EMPA, DAPA, and CANA had no effect on peak I_Na_ in the cardiomyocytes of an HF mouse model, but could significantly inhibit *I*_Na-Late_, which is a potent selective inhibitor of *I*_Na-Late_. Furthermore, EMPA reduced the incidence of spontaneous calcium transients induced by *I*_Na-Late_ (Philippaert et al., 2021). It also reduced the *I*_Na-Late_ of ventricular myocytes in diabetic rats compared with the control group (Lee et al., 2019). In mouse cardiomyocytes, EMPA, DAPA, and CANA reduced the activity of NHE and concentration of Na^+^ (Uthman et al., 2018; Uthman et al., 2019). Durak A et al. reported that there was a slight but significant increase in the maximum value of I_Na_ measured at -40mV with no change in voltage-dependency, whereas DAPA treatment restored this current in metabolic syndrome rats, significantly. But DAPA didn’t affect the intracellular Na^+^ (Durak et al., 2018). Another report showed that EMPA could inhibit NHE flux in the rabbit and rat cardiomyocytes under high-glucose environment, reduce Na^+^ and Ca^2+^ levels in the cardiomyocytes, and increase the Ca^2+^ levels in the mitochondria. Under non-high glucose environment, EMPA could also reduce NHE flux and the concentration of Na^+^ in cardiomyocytes (Baartscheer et al., 2017). It was also reported that DAPA inhibits the upregulation of NHE in mouse cardiofibroblasts exposed to lipopolysaccharides (LPS). This effect may be related to the activation of AMP-activated protein kinase (AMPK) (Ye et al., 2018). Further, similar phenomenon was observed in human cardiomyocytes, and EMPA’s NHE inhibition was comparable to that of the NHE inhibitor, cariporide (Trum et al., 2020). However, the NHE inhibitor cariporide could significantly reduce the activity of NHE in isolated rat ventricular myocytes. Conversely, EMPA did not affect the concentration of Na^+^ in cardiomyocytes within a wide concentration range. There was no evidence that EMPA could ameliorate the concentration of Na^+^ in cardiomyocytes by reducing the activity of NHE (Chung et al., 2021).

SGLT2i can also improve intracellular Ca^2+^ overload in cardiomyocytes (Arow et al., 2020; Byrne et al., 2020). SGLT2i ameliorated Ca^2+^ regulation of cardiomyocytes in diabetic mice by increasing SERCA2a activity, resulting in increased intracellular Ca^2+^ influx into the SR and decreased intracellular Ca^2+^ concentration (Hammoudi et al., 2017; Joubert et al., 2017; Goerg et al., 2021). Lee et al. found that EMPA could reduce the decay time of Ca^2+^ transients in ventricular myocytes of diabetic rats and reduce the incidence and frequency of Ca^2+^ spark. The duration and width of Ca^2+^ sparks also could be shortened by EMPA. SERCA2a activity was increased, RYR2 phosphorylation was lower, and intracellular Ca2^+^ concentration was decreased, but the reverse NCX current could be increased (Lee et al., 2019). EMPA also reduces CaMKII activity, thereby improving intracellular Ca^2+^ regulation in cardiomyocytes (Mustroph et al., 2018). A study showed that compared with the control group, DAPA didn’t change in both the maximum value and voltage-dependency of I_CaL_ measured at 0mV in metabolic syndrome rats’ cardiomyocytes. Rats’ cardiomyocytes treated with DAPA had larger averaged peak amplitude of Ca^2+^ transients, shorter time to peak amplitude and the half-time for recovery of Ca^2+^ transients, and more responses to acute caffeine (10 mM) exposures than in the metabolic syndrome rats’ cardiomyocytes (Durak et al., 2018).

**FIGURE 1:**
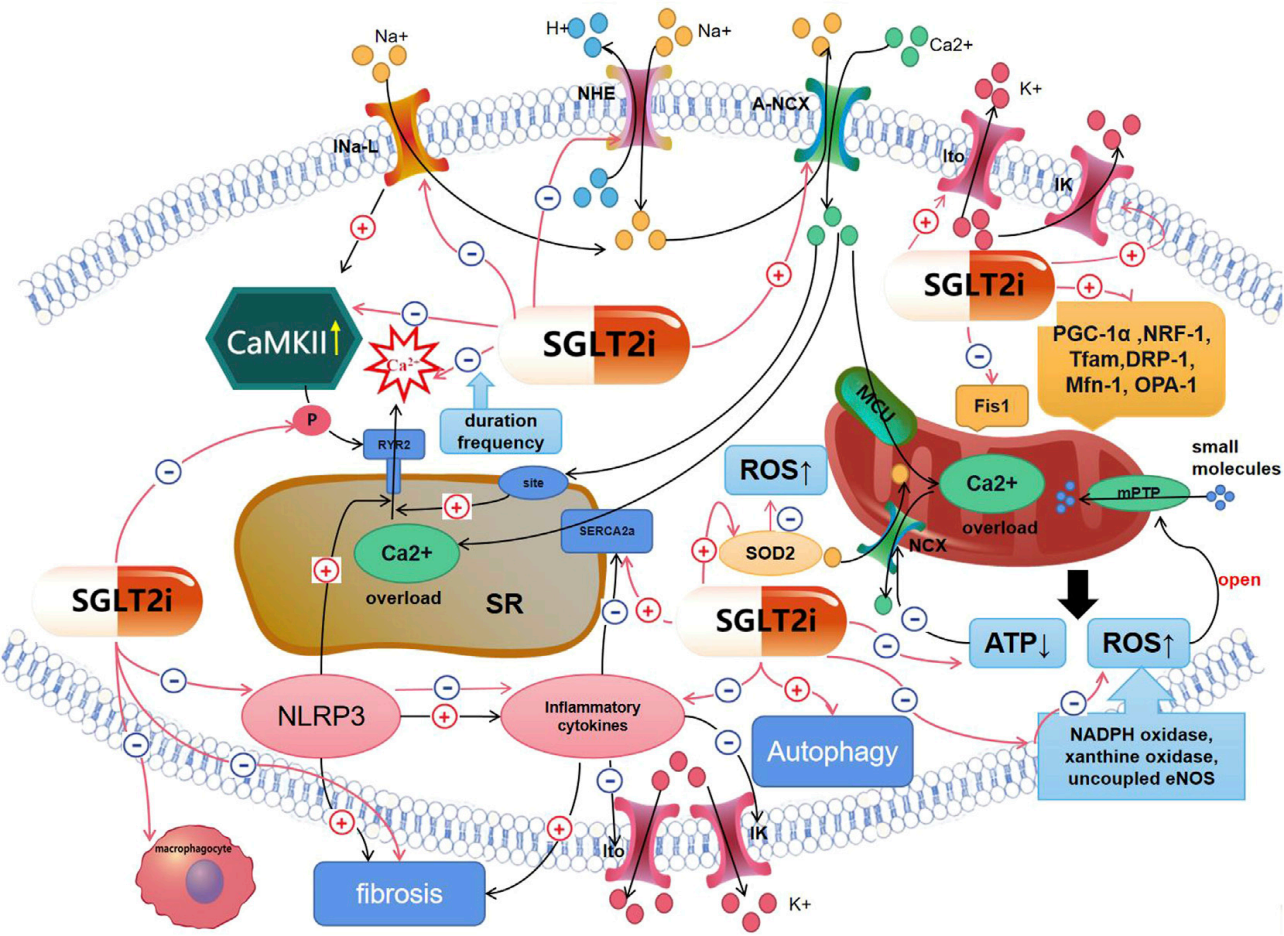
Mechanisms of SGLT2is′ effect on arrhythmias. Red lines represent the effect of SGLT2i; black lines mean mechanisms of arrhythmias. (1)SGLT2is can affect cardiomyocytes’ electrical remodeling, including inhibiting *I*_Na-Late_, *NHE*,*Ca* ^*2+*^ spark, the decreasing of ATP, RYR2 phosphorylation, CaMKII activity, inflammatory cytokines; increasing *Ito, IK,* SERCA2a activity, so that intracellular *Na* ^*+*^ and *Ca* ^*2+*^ overload and the disorder of *K* ^*+*^ currents could be ameliorated. (2)SGLT2is can ameliorate arrhythmias by inhibiting myocardial inflammation and structural remodeling, including inhibiting *NLRP3*, inflammatory cytokines and fibrosis, so that *K* ^*+*^ currents’ disorder can be improved. (3)SGLT2is can ameliorate arrhythmias by improving mitochondrial dysfunction and oxidative stress, including promoting mitochondrial biogenesis; inhibiting the decreasing of ATP, the increasing of ROS, so that intracellular ions administration can be improved. (4)SGLT2is can ameliorate arrhythmias by ameliorating autophagy.

SGLT2i can also ameliorate the disorder of K^+^ currents. Jhuo SJ et al. found that H9c2 cells were treated with adipocytokines from the pericardial and peripheral fat from the 20 C57BL/6J mice, divided into the control group, the metabolic syndrome group, the EMPA group, and the glibenclamide group, the I_K_ in the EMPA group was significantly higher than that in the metabolic syndrome group and the glibenclamide group in the metabolic syndrome mice model (Jhuo et al., 2020). Durak A et al. found that compared with metabolic syndrome rats, I_K_ in cardiomyocytes from metabolic syndrome rats treated with DAPA were found to be significantly increased at positive voltages such as from 0 to +70mV. Although the I_K_ at negative potentials did not change, DAPA induced a significant increase in I_K_ measured at -120mV. This effect of DAPA can be interpreted as its effect on the membrane potential to keep it at normal levels (Durak et al., 2018). However, there is no more relevant literature confirming that SGLT2is can improve the cardiomyocytes’ K^+^ currents currently (Figure 1).

### Inflammation, Cardiac Structural Remodeling, and Arrhythmias

Inflammation is classified as acute and chronic inflammation, which includes three pathological processes: metamorphism, exudation, and hyperplasia. Inflammation is an essential component of the healing process of damaged tissues. However, chronic inflammation occurs when damaged tissue do not heal in a short time, leading to pathological healing and permanent fibrosis at the damaged site (Passino et al., 2015). Cardiac inflammation mainly occurs in CVDs such as myocardial infarction, HF, and myocarditis and is closely related to the occurrence of arrhythmias (Ishii et al., 2005; Peretto et al., 2020). Inflammatory cytokines, such as TNF-α, IL-1, IL-6, IL-8, and chemokines, are indicators of the degree of inflammation. Meanwhile, inflammatory cytokines are related closely to **arrhythmias for** inducing cellular electrical remodeling. TNF-α and IL-1β are two important cytokines that mediate inflammation and are mainly produced by M1-like macrophages. Saba S et al. found that compared with normal mice, the TNF-α mice (TNF1.6) with the overexpression of TNF-α are prone to spontaneous atrial arrhythmias. Compared with control atrial myocytes, female and male TNF1.6 myocytes displayed a significant reduction in the amplitude, the +dCa_i_^2+^/dt_max_ and -d Ca_i_^2+^/dt_max_ of the Ca_i_^2+^ transient, and prolongation of the TD_50%_ (Saba et al., 2005). Fernández-Velasco M et al. found that compared with control, ventricular myocytes from Wistar rats treated with TNF-α showed significantly prolonged APD and reduced I_to_, including amplitude and density. This manifestation may be caused by the selective inducible nitric oxide synthase (iNOS) induction and generation of oxidant species. But the I_CaL_ didn’t change (Fernández-Velasco et al., 2007). Monnerat G demonstrated that in the ventricular cardiomyocytes treated with IL-1β, the APD was prolonged, the density of Ito was reduced, the amplitude and frequency of Ca^2+^ spark increased, but the SR Ca^2+^ content, the rate constants and the ratio of Ca^2+^ re-uptake/efflux were not significantly changed. Meanwhile, IL-1β significantly enhanced the number of spontaneous contractile events (NSE) in ventricular myocytes, and the effect of IL-1β on NSE was weakened in cardiomyocytes from mice with genetic CaMKII inhibition by cardiac-specific expression of autocamtide 3 inhibitory peptide (Monnerat et al., 2016). The nucleotide-binding oligomerization domain NOD-like receptor 3 (NLRP3), a molecular switch regulating inflammation, promotes the production of IL-1β and IL-8 in ischemic cardiomyocytes (Zhou et al., 2020a), inducing more serious inflammation. NLRP3 can further cause SR Ca^2+^ leakage and intracellular Ca^2+^ overload (Murakami et al., 2012). TNF-α is also closely related to the occurrence of cardiac electrical remodeling (London et al., 2003; Li et al., 2020). In rabbit pulmonary vein cardiomyocytes treated with TNF-α, a significant increase was observed in DADs amplitude, with a longer attenuation of the calcium transient and reduced expression levels of SERCA2a as compared to controls (Lee et al., 2007).

Inflammation can also lead to structural cardiac remodeling. Reportedly, the amount of total left atrial collagen in patients with AF is positively correlated with the level of inflammatory cytokines, including TNF-α, IL-6, MCP-1, and MMP-9 (Abe et al., 2018). Inflammatory cytokines activate immune cells, particularly monocytes, to recruit them from circulation into the myocardium. Once monocytes enter the myocardium, they differentiate into macrophages, which promote inflammation, damage, and fibrosis of the myocardium (Passino et al., 2015; Mack, 2018). In addition, macrophages also play an important role in the conduction of the cardiomyocytes. And connexin 43 (CX43) is crucial for macrophages’ conduction, and its abnormal expression and distribution will lead to the abnormality of the conduction of the cardiomyocytes, decreasing the conduction velocity and changing the anisotropy, producing arrhythmias (Peters et al., 1997; Morel et al., 2012; Zhang et al., 2014). Hulsmans et al. found that elongated cardiac macrophages expressing CX43, interspersing in the distal atrioventricular node, connecting with cardiomyocytes through CX43, facilitate electrical conduction, whereas conditional deletion of CX43 in macrophages and congenital lack of macrophages delay atrioventricular conduction (Hulsmans et al., 2017). Myocardial fibrosis is a sign of cardiac structural remodeling and the basis for the occurrence and persistence of arrhythmias, especially AF (Dzeshka et al., 2015; Moreira et al., 2020). As cardiomyocytes are permanent and non-regenerative, regeneration occurs via cells such as the interstitial cells, macrophages, and fibroblasts, during the hyperplasia phase. Fibroblasts play an important role in myocardial fibrosis development and are non-excitable cells in nature. However, they transmit electricity between cardiomyocytes via connexins, resulting in inhomogeneous conduction, depolarization of resting cardiomyocytes, and induced automatic depolarization of the four phases (Sohns and Marrouche, 2020), thereby increasing the risk of arrhythmias. Transforming growth factor beta-1 (TGF-β1), an effective stimulator of fibroblasts, has a pathogenic effect on valvular heart disease and arrhythmias (Khan and Sheppard, 2006; Salvarani et al., 2017; Liu et al., 2019). In addition, angiotensin II (Ang II) can also lead to myocardial fibrosis and increase the probability of arrhythmia (Purohit et al., 2013; Wang et al., 2019).

### SGLT2i can Ameliorate Myocardial Inflammation and Structural Remodeling

SGLT2i have been reported to ameliorate inflammation (Lee et al., 2021; Wang et al., 2021). EMPA is shown to inhibit the increase of TNF-α and IL-6 levels in the cardiac tissue of Zucker diabetic fatty rats and patients with heart failure with preserved ejection fraction (HFpEF), and ameliorate microvascular inflammation (Kolijn et al., 2021). In *vitro* assays, it decreased the expression levels of TNF-α in LPS-induced mouse atrial myocytes and increased the expression levels of an anti-inflammatory M2 marker protein in LPS-treated macrophages (Koyani et al., 2020). EMPA also inhibits the infiltration of myocardial macrophages exposed to excessive glucocorticoids for a long time (Zhang et al., 2020). Philippaert et al. found that EMPA or tetrodotoxin infusion in isolated mouse hearts prevented the activation of NLRP3 in cardiomyocytes (Philippaert et al., 2021). It also reduced the mRNA levels of IL-6, chemokines, TNF-α, and MCP-1 in cardiomyocytes of diabetic rats significantly (Aragón-Herrera et al., 2019). The expression levels of IL-1β, IL-8, IL-6, and NLRP3 in mouse cardiomyocytes co-incubated with doxorubicin (DOXO)-EMPA were significantly reduced compared with those in mouse cardiomyocytes co-incubated with DOXO (Quagliariello et al., 2021). In addition, DAPA significantly reduced the mRNA levels of IL-1β, IL-6, NLRP3, and TNF-α in cardiomyocytes of T2MD mice (Ye et al., 2017). The anti-inflammatory effects of EMPA have also been observed in the kidneys and liver (Elkazzaz et al., 2021; Nasiri-Ansari et al., 2021).

SGLT2i can improve cardiac structural remodeling (Chowdhury et al., 2020; Madonna et al., 2020; Sun et al., 2020; Shentu et al., 2021). EMPA was reported to affect the oxidative stress and fibrosis by inhibiting the TGF-β/Smad signaling pathway (Li et al., 2019; Daud et al., 2021) and activate the Nrf2/ARE signaling pathway in cardiomyocytes in the T2MD mouse model (Li et al., 2019). DAPA also inhibits fibroblast activation and myocardial fibrosis (Shih et al., 2021; Tian et al., 2021) by inhibiting the TGF-β/Smad signaling pathway (Tian et al., 2021) in T2MD rats. EMPA could shift myocardial fuel from glucose to ketone bodies, free fatty acids, and branched amino acids to ameliorate the myocardial energetics and left ventricular structural remodeling of the non-diabetic HF pig model. A reduction in myocardial fibrosis was observed in imaging, histological, and cytological assessments (Santos-Gallego et al., 2019; Santos-Gallego et al., 2021). Sabatino et al. also found that, compared with the DOXO group, the degree of myocardial fibrosis in mice was reduced by 50% in the DOXO-EMPA group (Sabatino et al., 2020). DAPA is also shown to reduce the infiltration of myocardial fibroblasts and fibrosis after myocardial infarction in mice (Lee et al., 2017). The levels of collagen-1 and collagen-3 mRNA in the left ventricle of T2MD mice were also decreased by DAPA (Ye et al., 2017). It could inhibit myocardial hypertrophy, myocardial fibrosis, and increase myocardial collagen in SD rats injected with Ang II (Zhang et al., 2021). However, DAPA had a small effect on improving myocardial hypertrophy and fibrosis in the HFpEF model (Withaar et al., 2021). Meanwhile, a study showed compared with the metabolic group, significant attenuation of downregulated connexins (connexin40 and CX43) expression and significantly fewer fibrotic areas in ventricles of metabolic mice treated with EMPA, and the ECG QT interval was significantly shorter. The ERP of the left ventricle was also significantly shorter in the EMPA group than that in the control, metabolic, and glibenclamide groups (Jhuo et al., 2021)(Figure 1).

### Mitochondrial Dysfunction, Oxidative Stress, and Arrhythmias

Mitochondria are energy factories that produce adenosine triphosphate (ATP). ATP production is achieved by the mitochondrial membrane potential (ΔΨ). ΔΨ is a criterion for evaluating the function of mitochondria, which can generate the necessary proton propulsion for ATP generation and release the energy required to phosphorylate ADP to ATP (Mitchell, 1961; El Hadi et al., 2019). Mitochondrial dysfunction is closely related to arrhythmias (Lin et al., 2003; Ahmad et al., 2018; Saadeh et al., 2021). When mitochondrial function is impaired, ATP production is hampered, such that the function of Na^+^ and Ca^2+^ pumps on the cell membrane is inhibited, concentration of Na^+^ and Ca^2+^ in the cardiomyocyte cytoplasm is overloaded, and normal electrical activity of cardiomyocytes is disrupted, resulting in arrhythmias (Gambardella et al., 2017). Overloaded Ca^2+^ can enter mitochondria through MCU, but due to the decrease in ATP production, NCX dysfunction occurs, resulting in an imbalance of Ca^2+^ flow in and out of the mitochondria. This imbalance results in the accumulation of Ca^2+^ in the mitochondria. The increased Ca^2+^ levels cause mitochondrial swelling, damage to ΔΨ and the respiratory chain, and further reduction of ATP generation, leading to arrhythmias (Boyman et al., 2021; Pool et al., 2021). Mitochondrial division/fusion and biogenesis play important roles in the maintenance of mitochondrial homeostasis. Peroxisome proliferator-activated receptor-γ co-activator-1α (PGC-1α) is a critical regulator of mitochondrial biosynthesis that promotes mitochondrial biogenesis and plays an important role in transcriptional regulation (Gleyzer et al., 2005). PGC-1α can regulate the expression and phosphorylation of mitochondrial fission/fusion proteins mitofusin 2 (Mfn2) and dynamin-related protein 1 (Drp1) to maintain the balance between mitochondrial fission and fusion (Peng et al., 2017), alleviate mitochondrial overdecomposition, and improve mitochondrial fusion levels. The mitochondrial outer membrane fusion is mediated by the membrane-localized GTPase mitofusins Mfn1 and Mfn2, and the intimal fusion is mediated by the membrane-localized GTPase OPA1. When mitochondria are dysfunctional, fission mediated by Drp1 and mitochondrial fission 1 (Fis1) can be promoted (Chen and Chan, 2010).

The mitochondrial respiratory chain is a major component of the reactive oxygen species (ROS) production in the body. NADPH oxidase, xanthine oxidase, and uncoupled endothelial nitric oxide synthase (eNOS) have been identified as the main sources of ROS. Under physiological conditions, ROS is produced in small amounts and can be removed by the antioxidant system over time. Under pathological conditions, ROS production is increased. When a large amount of ROS cannot be removed by the antioxidant system in time, oxidative stress occurs, resulting in the degeneration of proteins, lipids, and nucleic acids, and the normal structure and function of cells is destroyed (Zorov et al., 2014; Senoner and Dichtl, 2019). When mitochondria are dysfunctional, ROS production increases, which can further promote mitochondrial production of a large amount of ROS, a process known as ROS-induced ROS release (Zorov et al., 2000). Increased ROS levels result in loss of ion channel function and ion disorder in cardiomyocytes, leading to an increased probability of arrhythmia (Liu et al., 2010; Sovari and Dudley, 2012). In addition, ROS can further allow the mitochondrial permeability transition pore to open continuously, allowing large numbers of small molecules to enter into the mitochondria at will. Therefore, when ΔΨ is damaged, ATP production is reduced, calcium dyshomeostasis occurs, and the cell dies, stimulating further ROS production and thus promoting the occurrence of arrhythmias (Pérez and Quintanilla, 2017; Gordan et al., 2020). PGC-1α also plays a regulatory role in ROS clearance (Bai et al., 2011).

### SGLT2i can Ameliorate Mitochondrial Dysfunction and Oxidative Stress

In recent years, studies have demonstrated that SGLT2i can improve mitochondrial dysfunction. The activation of AMPK mediated by EMPA reduced the consumption of ATP/ADP in cardiomyocytes cultured with LPS *in vitro* (Koyani et al., 2020). It also ameliorated the decrease of ATP levels in the non-infarct area after myocardial infarction in diabetic mice (Oshima et al., 2019). In atrial myocytes of diabetic mice model, high-dose EMPA (30 mg/kg) significantly improved mitochondrial respiratory function and ΔΨ. This dose led to increase in the expression levels of PGC-1α, NRF-1, Tfam, DRP-1, Mfn-1, and OPA-1. However, low-dose EMPA (10 mg/kg) did not improve DRP-1 significantly (Shao et al., 2019). EMPA also affected the upregulation of Fis1 and oxidative stress and recovered autophagy in the non-infarct area after myocardial infarction in diabetic mice, thus rescuing the mitochondrial numbers and size (Mizuno et al., 2018). In the rat model of cardiac ischemia/reperfusion (I/R), DAPA was more effective in reducing mitochondrial fission as compared to the dipeptidyl peptidase 4-inhibitor vildagliptin (Tanajak et al., 2018).

SGLT2i ameliorates oxidative stress. EMPA has been shown to reduce the oxidative stress by increasing the expression levels of superoxide dismutase 2, the antioxidant enzyme, in the myocardial tissue of diabetic mice (Li et al., 2019). It could also inhibit the activity of NADPH oxidase, significantly reducing the content of NADPH oxidase subtypes NOX1 and NOX2 in aortic cells of type I diabetes mellitus model rats. Further, it was able to reduce the levels of oxidative protein, increase the REDOX-sensitive enzyme aldehyde dehydrogenase 2 levels, significantly reduce the generation of ROS in aortic cells, and dose-dependently reduce the expression levels of eNOS and dihydrofolate reductase (Oelze et al., 2014). In addition, EMPA increased cardiac GTP enzyme cyclohydrolase 1 in cardiomyocytes of diabetic myocardial infarction model rats, thereby reducing ROS production (Asensio Lopez et al., 2020). It also inhibited the gene expression of inducible NO synthase in RAW 264.7 mouse macrophages post induction by LPS (Lee et al., 2021). Durak A et. also found that DAPA could normalize the levels of oxidative stress in cardiomyocytes of rats with metabolic syndrome, thereby inhibiting the prolonged ventricular-repolarization (Durak et al., 2018) (Figure 1).

### Autophagy and Arrhythmias

Autophagy, also known as type II programmed cell death, is a lysosome-dependent catabolic pathway, which can be caused by an increase in AMPK activity and decrease in mammalian target of rapamycin (mTOR) activity. It can also be induced by separation of the Beclin protein complex from Bcl-2 (Mizushima et al., 2008; Alers et al., 2012; Jiang et al., 2018; Xu and Qin, 2019). Autophagy plays an important role in maintaining homeostasis (Parzych and Klionsky, 2014). During myocardial ischemia, the fusion of autophagosomes and lysosomes is blocked and autophagosome flow is impaired, leading to the accumulation of autophagosomes. This loss of function of clearing damaged organelles results in oxidative stress injury, ROS accumulation, and increased mitochondrial permeability (Shi et al., 2019), thereby increasing the risk of arrhythmias (Wiersma et al., 2017). In I/R rat model, the autophagy biomarkers Beclin-1 and LC3B-II/LC3B-I were significantly higher in the cardiomyocytes of VF mice than in those of non-VF mice (Meyer et al., 2013). Autophagy is also reduced in patients with AF. Studies have shown that in patients with cardiac valvular disease, the expression levels of autophagy markers, LC3B-II and LC3B-II/LC3B-I, were significantly reduced in patients with AF compared with those in patients with sinus rhythm (Chen et al., 2011; Zhou et al., 2020b).

### SGLT2i can Ameliorate Autophagy

SGLT2i can ameliorate autophagy by upregulating mitochondrial autophagy-related protein Bnip3 mRNA levels, thereby increasing Bnip3 protein levels and preventing a decrease in the number of autophagy vacuoles in diabetic myocardial infarction model mice (Mizuno et al., 2018). EMPA and DAPA activated AMPK in the cardiomyocytes (Joubert et al., 2017; Aragón-Herrera et al., 2019) and enhance autophagy in early diabetic rats (Aragón-Herrera et al., 2019). Additionally, the effect of SGLT2i on improving the autophagy response has also been demonstrated in the liver and kidney. EMPA was found to enhance hepatic macrophage autophagy by enhancing the AMPK/mTOR signaling pathway, thereby inhibiting the IL-17/IL-23 inflammatory axis, alleviating inflammation, and significantly improving liver injury in T2DM mice with non-alcoholic fatty liver disease (NAFLD) (Meng et al., 2021). Nasiri-Ansari N et al. reported that EMPA could increase the AMPK/mTOR signaling pathway in NAFLD mice. Moreover, they found that the expression levels of autophagy markers LC3B and Bcl2/Bax was upregulated, leading to increase in autophagy in liver cells (Nasiri-Ansari et al., 2021). EMPA also regulates autophagy in glomerular podocytes. It was found that EMPA could increase the volume density of autophagosomes, autophagolysosomes, and lysosomes. Coupled with an increase in the levels of key regulatory protein Beclin-1 and the autophagy marker LAMP-1, it increased the apoptosis marker Bcl-2 levels in db mouse glomerular podocytes. When EMPA was combined with linagliptin, the expression levels of the apoptotic marker caspase-3 were reduced (Korbut et al., 2020) (Figure 1).

## Conclusion

SGLT2i are a new type of hypoglycemic drug with good safety and tolerance. It can regulate blood glucose levels safely and effectively, thereby reducing the occurrence and progression of heart failure and cardiovascular events significantly. Studies have confirmed that SGLT2i ameliorates arrhythmia, but the specific mechanism remains unclear. It may ameliorate the occurrence and development of arrhythmias by improving cardiac electrical remodeling, mitochondrial dysfunction, cardiac structural remodeling, inflammation, oxidative stress, and autophagy. However, there is a lack of relevant studies to directly prove that SGLT2i can improve arrhythmias by improving the above-mentioned factors. A clinical trial to explore the related mechanisms of SGLT2i in ameliorating arrhythmias is required. This can aid in understanding the mechanisms in play and provide effective guidance for the drug treatment of arrhythmias in clinical practice.
